# Complete plastome sequence of *magnolia fordiana* var. *hainanensis* (dandy) Noot. (Magnoliaceae), an endemic and ornamental tree in South China

**DOI:** 10.1080/23802359.2019.1699456

**Published:** 2019-12-13

**Authors:** Yu-Wen Zou, Hong-Xin Wang, Zhi-Xin Zhu, Hua-Feng Wang

**Affiliations:** Key Laboratory of Tropical Biological Resources of Ministry of Education, School of Life and Pharmaceutical Sciences, Hainan University, Haikou, China

**Keywords:** *Magnolia fordiana* var. *hainanensis*, plastome, phylogeny, genome structure, magnoliaceae

## Abstract

*Magnolia fordiana* var. *hainanensis* (Dandy) Noot. (Magnoliaceae) is an endemic and ornamental tree distributed in Hainan, China. In this study, we report and characterize the complete plastid genome sequence of *M. fordiana* var. *hainanensis* in order to provide genomic resources helpful for promoting its conservation and garden utilization. The complete plastome is 160,157 bp in length and contains the typical quadripartite structure of angiosperm, including two Inverted Repeat (IRs) regions of 26,573 bp, a Large Single-Copy (LSC) region of 88,255 bp and a Small Single-Copy (SSC) region of 18,756 bp. The plastome contains 114 genes, consisting of 80 unique protein-coding genes, 30 unique tRNA gene and 4 unique rRNA genes. The overall A/T content in the plastome of *M. fordiana* var. *hainanensis* is 60.70%. The complete plastome sequence of *M. fordiana* var. *hainanensis* will provide a useful resource for the conservation and garden utilization of this species as well as for the phylogenetic studies of Magnoliaceae.

## Introduction

*Magnolia fordiana* var. *hainanensis* (Dandy) Noot. was an evergreen tree belonging to Magnoliaceae. Its height and trunk diameter could reach to 20 m and 45 cm respectively. Leaf blade is thinly leathery and leaf margin is wavy. Peduncle is glabrous. It could be used as a good ornamental plant for its showy white flowers. *Magnolia fordiana* var. *hainanensis* is native to Hainan, China and distributed in 300-1, 200 m (China ECoFo 2013). Consequently, the genetic and genomic information is urgently needed to promote its systematics research and the development of conservation value of *M. fordiana* var. *hainanensis.* In this study, the complete plastome of *M. fordiana* var. *hainanensis* (GenBank accession number: MN306583) was reported and characterized. This is the first report of a complete plastome for *M. fordiana* var. *hainanensis.*

In this study, *M. fordiana* var. *hainanensis* was sampled from Diaoluo Mountain (18.67 N, 109.88E), which is a National Nature Reserve of Hainan, China. A voucher specimen (Wang et al. B55) was deposited in the Herbarium of the Institute of Tropical Agriculture and Forestry (HUTB), Hainan University, Haikou, China.

The experiment procedure is as reported in Zhu et al. (2018). Around 6 Gb clean data were assembled against the plastome of *Magnolia tripetala* (NC_024027.1) (Zhu et al. 2016) using MITObim v1.8 (Hahn et al. 2013). The plastome was annotated using Geneious R8.0.2 (Biomatters Ltd., Auckland, New Zealand) against the plastome of *M. tripetala* (NC_024027.1). The annotation was corrected with DOGMA (Wyman et al. 2004).

The plastome of *M. fordiana* var. *hainanensis* is found to possess a total length 160,157 bp with the typical quadripartite structure of angiosperms, contains two Inverted Repeats (IRs) of 26,573 bp, a Large Single-Copy (LSC) region of 88,255 bp and a Small Single-Copy (SSC) region of 18,756 bp. The plastome contains 114 genes, consisting of 80 unique protein-coding genes, 30 unique tRNA genes and 4 unique rRNA genes. The overall A/T content in the plastome of *M. fordiana* var. *hainanensis* is 60.70%, which the corresponding value of the LSC, SSC and IR region were 62.00%, 65.80% and 56.80%, respectively.

**Figure 1.**
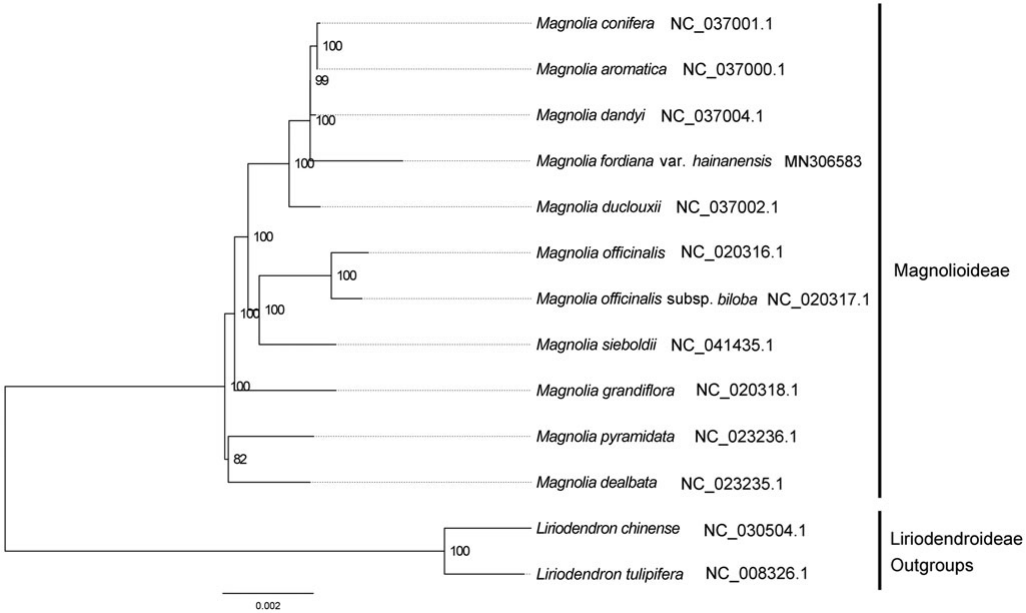
The best ML phylogeny recovered from 13 complete plastome sequences by RAxML. Accession numbers: *Magnolia fordiana* var. *hainanensis* MN306583, *Magnolia aromatica* NC_037000.1, Magnolia conifera NC_037001.1, *Magnolia dandyi* NC_037004.1, *Magnolia dealbata* NC_023235.1, *Magnolia duclouxii* NC_037002.1, *Magnolia grandiflora* NC_020318.1, *Magnolia officinalis* NC_020316.1, *Magnolia officinalis* subsp. *biloba* NC_020317.1, *Magnolia pyramidata* NC_023236.1, *Magnolia sieboldii* NC_041435.1. Outgroups: *Liriodendron chinense* NC_030504.1*, Liriodendron tulipifera* NC_008326.1.

We used RAxML (Stamatakis 2006) with 1,000 bootstraps under the GTRGAMMAI substitution model to reconstruct a maximum likelihood (ML) phylogeny of 10 published complete plastomes of Magnolioideae, using 2 species of Liriodendroideae as outgroups. The phylogenetic analysis indicated that *M. fordiana* var. *hainanensis* is close to *Magnolia dandyi* within Magnoliaceae in this study (Figure 1). Most nodes in the plastome ML tree were strongly supported. The complete plastome sequence of *M. fordiana* var. *hainanensis* will provide a useful resource for the conservation genetics of this species as well as for the phylogenetic studies of Magnoliaceae.
